# P-1566. Healthcare Resource Use and Costs of Acute Uncomplicated Cystitis in Japan

**DOI:** 10.1093/ofid/ofae631.1733

**Published:** 2025-01-29

**Authors:** Meg Franklin, Maia R Emden, Elise Bauer, Naomi Sacks, Fanny S Mitrani-Gold, Shinyoung Ju, Ashish V Joshi, Yoshiaki Kawano, Shinya Kawamatsu, Madison T Preib

**Affiliations:** PRECISIONheor, Boston, MA, USA; Franklin Pharmaceutical Consulting, Cary, NC, USA, Boston, Massachusetts; PRECISIONheor, Boston, Massachusetts; PRECISIONheor, Boston, Massachusetts; PRECISIONheor, Boston, Massachusetts; GlaxoSmithKline plc., Collegeville, Pennsylvania; GSK, Brentford, England, United Kingdom; GlaxoSmithKline plc., Collegeville, Pennsylvania; GSK, Brentford, England, United Kingdom; GSK, Brentford, England, United Kingdom; GSK, Brentford, England, United Kingdom

## Abstract

**Background:**

Acute uncomplicated cystitis (AUC) is an area of interest for outpatient antimicrobial stewardship in Japan due to the high prevalence of community antibiotic use. Literature on the cost burden of AUC in Japan is limited. We aimed to quantify real-world healthcare resource utilization (HCRU) and costs of treating AUC in Japan.

**Figure:**
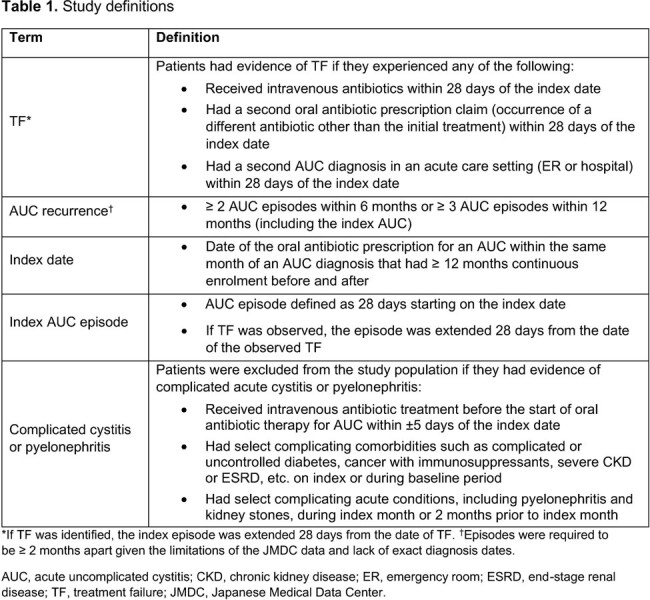

**Methods:**

This retrospective cohort study used Japanese Medical Data Center (JMDC) claims data (1 October 2015−30 November 2021). Eligible patients (≥ 18 to < 75 years) had an outpatient AUC diagnosis claim in the same month as ≥ 1 oral antibiotic prescription (date of prescription = index date). Continuous health plan enrolment, with medical and pharmacy benefits, was required ≥ 12 months pre index (baseline period) and ≥ 12 months post index (follow-up period). Patients with complicated cystitis or pyelonephritis were excluded.

The index AUC episode was defined as 28 days from index date and was extended an additional 28 days if treatment failure (TF) was observed (within 28 days from date of TF). Patients were stratified based on evidence of TF or AUC recurrence (full definitions in **Table 1**). AUC-related costs (Japanese Yen [¥]) and HCRU, per episode, were compared within subgroups.

**Figure:**
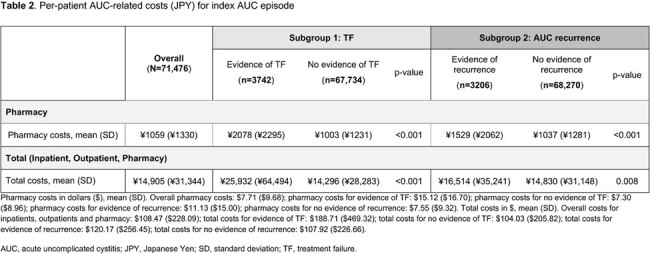

**Results:**

Overall, 71,467 patients were included, of whom 3742 (5%) had evidence of TF and 3206 (4%) had recurrent AUC. The mean (standard deviation [SD]) cost of any AUC episode was ¥14,905 (¥31,344) (**Table 2**), with 1.22 (0.46) outpatient claims and 1.07 (0.27) pharmacy prescription claims per episode (**Table 3**). Patients with evidence of TF had significantly higher mean (SD) pharmacy (¥2078 [¥2295]) and total costs (¥25,932 [¥64,494]), and a higher number of pharmacy prescription claims (2.03 [0.45]) per episode versus patients without TF (all p< 0.001). Patients with recurrent AUC had higher mean (SD) total costs (¥16,514 [¥35,241]) and higher outpatient claims (1.10 [0.34]) per episode versus patients without recurrence (¥14,830 [¥31,148]) total costs and (¥1037 [¥1281]) outpatient claims; (p=0.008 and p< 0.001, respectively).

**Figure:**
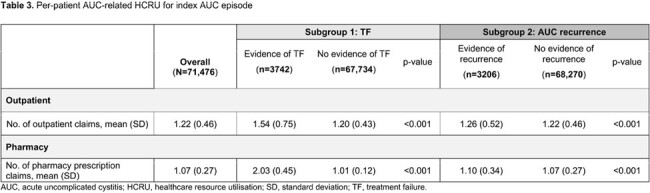

**Conclusion:**

TF rates and recurrence of AUC in Japan were modest; however, the economic burden of AUC was greater in patients who had evidence of TF or recurrence.

**Disclosures:**

**Meg Franklin, PharmaD, PhD**, Franklin Pharmaceutical Consulting: Owner and President|Franklin Pharmaceutical Consulting: Ownership Interest|PRECISIONheor (who received funding from GSK to complete this study): Advisor/Consultant **Maia R. Emden, BA**, PRECISIONheor: Previous employee of PRECISIONheor, who received funding from GSK to complete this study **Elise Bauer, MS**, PRECISIONheor: Previous employee of PRECISIONheor, who received funding from GSK to complete this study **Naomi Sacks, PhD**, PRECISIONheor: Previous employee of PRECISIONheor, who received funding from GSK to complete this study **Fanny S. Mitrani-Gold, MPH**, GSK: Employee|GSK: Stocks/Bonds (Public Company) **Shinyoung Ju, MS**, GSK: Employee|GSK: Stocks/Bonds (Public Company) **Ashish V. Joshi, PhD**, GSK: Employee|GSK: Stocks/Bonds (Public Company) **Yoshiaki Kawano, MD, PhD**, GSK: Employee|GSK: Stocks/Bonds (Public Company) **Shinya Kawamatsu, PhD**, GSK: Employee|GSK: Stocks/Bonds (Public Company) **Madison T. Preib, MPH**, GSK: Employee|GSK: Stocks/Bonds (Public Company)

